# Supplementary material for: Effects of Temperature and Density on House Cricket Survival and Growth and on the Prevalence of Acheta Domesticus Densovirus
Source: Insects. 2023 Jun 29;14(7):588. doi: 10.3390/insects14070588 (PMC10380462; doi:10.3390/insects14070588)
Supplement: Supplementary file 1 [file insects-14-00588-s001.zip › insects-2419124-supplementary.pdf]

Supplementary figures

Table S1. Analysis of variance (ANOVA) output tables for the three experimental runs on relative viral abundance data.

Experimental run 1	Analysis of variance				
	Df	SS	MS	F	p-value
Temp	2	12.87	6.437	3.509	0.0405
Dens	2	0.9	0.448	0.244	0.7845
Temp:Dens	4	12,73	3.184	1.736	0.1635
Residuals	36	66.03	1.834		
**Experimental run 2**	**Analysis of variance**				
	Df	SS	MS	F	p-value
Temp	2	0.61	0.306	0.176	0.8391
Dens	2	5.67	2.835	1.635	0.2092
Temp:Dens	4	15.38	3.845	2.217	0.0866
Residuals	36	62.44	1.734		
**Experimental run 3**	**Analysis of variance**				
	Df	SS	MS	F	p-value
Temp	2	25.34	12.671	7.151	0.00243
Dens	2	22.16	11.080	6.253	0.00467
Temp:Dens	4	5.04	1.259	0.710	0.59016
Residuals	36	63.79	1.772		

Table S2. P values for the pairwise comparisons for the biomass data.

P =	25C° D10	25C° D20	25C° D40	30C° D10	30C° D20	30C° D40	35C° D10	35C° D20	35C° D40
25C° D10		<.0001	<.0001	0.0002	<.0001	<.0001	0.0004	<.0001	<.0001
25C° D20			<.0001	<.0001	0.0098	<.0001	<.0001	0.0375	<.0001
25C° D40				<.0001	<.0001	0.1112	<.0001	<.0001	0.4317
30C° D10					<.0001	<.0001	1.0000	<.0001	<.0001
30C° D20						<.0001	<.0001	1.0000	<.0001
30C° D40							<.0001	<.0001	0.9988
35C° D10								<.0001	<.0001
35C° D20									<.0001

Table S3. Survival, harvested biomass and individual weight of house crickets reared at nine combinations of temperatures and densities.

Treatment	Survival (%) (±SE)	Mean harvested biomass (mg) (±SE)	Mean female weight (mg) (±SE)	Mean male weight (mg) (±SE)
25C° D10	79.3 (±7)	2114.467 (±5)	273 (±7.22)	246 (±6.23)
25C° D20	83.0 (±5)	4442.133 (±4.29)	294 (±6.57)	241 (±4.42)
25C° D40	83.3 (±4)	8834.267 (±2.85)	283 (±4.12)	244 (±3.4)
30C° D10	81.3 (±7)	2832.400 (±7.59)	389 (±10.88)	315 (±8.69)
30C° D20	78.3 (±5)	5553.400 (±5.71)	400 (±7.44)	308 (±6.26)
30C° D40	77.0 (±4)	10491.133(±4.02)	391 (±6.44)	301 (±3.53)
35C° D10	84.7 (±8)	2785.933 (±5.55)	359 (±8.02)	302 (±6.02)
35C° D20	82.7 (±5)	5408.000 (±4.4.)	364 (±5.21)	279 (±4.3)
35C° D40	79.0 (±4)	10065.467 (±3.56)	351 (±4.66)	270 (±3.45)

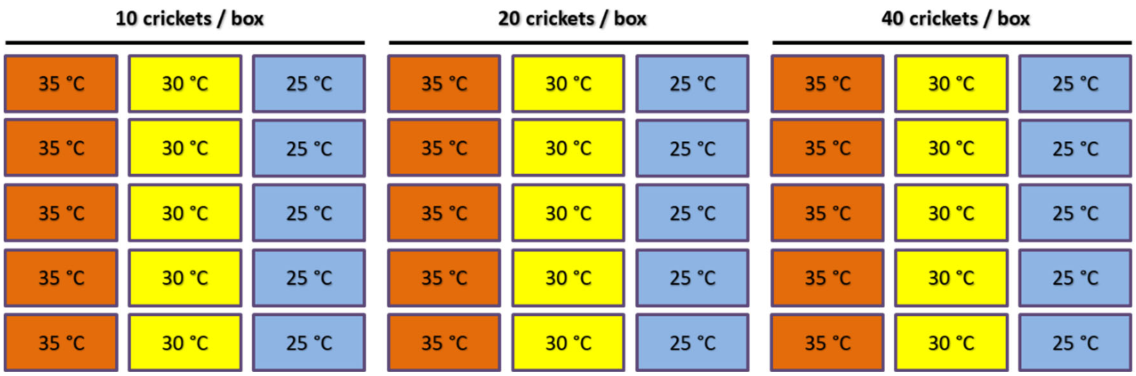

Figure S1. Overview of a single experimental run

Table S4.: Pairwise comparison data for female body weight.

P =	25C° D10	25C° D20	25C° D40	30C° D10	30C° D20	30C° D40	35C° D10	35C° D20	35C° D40
25C° D10		0.5244	0.9350	<0.0001	<0.0001	<0.0001	<0.0001	<0.0001	<0.0001
25C° D20			0.9548	<0.0001	<0.0001	<0.0001	<0.0001	<0.0001	<0.0001
25C° D40				<0.0001	<0.0001	<0.0001	<0.0001	<0.0001	<0.0001
30C° D10					0.9940	1.0000	0.2323	0.3979	0.0100
30C° D20						0.9644	0.0038	0.0025	<0.0001
30C° D40							0.0329	0.0293	<0.0001
35C° D10								0.9983	0.9999
35C° D20									0.7292

Table S5.: Pairwise comparison data for male body weight.

P =	25C° D10	25C° D20	25C° D40	30C° D10	30C° D20	30C° D40	35C° D10	35C° D20	35C° D40
25C° D10		1.0000	1.0000	<0.0001	<0.0001	<0.0001	<0.0001	0.0045	0.0740
25C° D20			1.0000	<0.0001	<0.0001	<0.0001	<0.0001	<0.0001	0.0001
25C° D40				<0.0001	<0.0001	<0.0001	<0.0001	<0.0001	<0.0001
30C° D10					0.9963	0.5928	0.9775	0.0008	<0.0001
30C° D20						0.9475	1.0000	0.0024	<0.0001
30C° D40							0.9998	0.0178	<0.0001
35C° D10							<0.0001	0.0661	0.0002
35C° D20									0.8375
